# Dentistry – a professional contained career in healthcare. A qualitative study of Vocational Dental Practitioners' professional expectations

**DOI:** 10.1186/1472-6831-7-16

**Published:** 2007-11-16

**Authors:** Jennifer E Gallagher, Wendy Clarke, Kenneth A Eaton, Nairn HF Wilson

**Affiliations:** 1 King's College London Dental Institute at Guy's, King's College and St Thomas' Hospitals, Oral Health Services Research & Dental Public Health, London, UK; 2 Formerly in King's College London Dental Institute at Guy's, King's College and St Thomas' Hospitals, Oral Health Services Research & Dental Public Health, London, UK; 3 Faculty of General Dental Practice (UK), Royal College of Surgeons of England, UK; 4 King's College London Dental Institute at Guy's, King's College and St Thomas' Hospitals, Office of the Dean and Head of School, London, UK

## Abstract

**Background:**

New graduates in the UK presently spend one year in training as Vocational Dental Practitioners (VDPs) in preparation for primary dental care. There is a growing recognition that the emerging workforce has very different professional expectations to those of earlier generations, with implications for the profession, patients and the performance of health systems. The objectives of this study were to investigate why VDPs' in England and Wales perceive they chose dentistry as a professional career; how they perceive their vision has changed and the implications for their professional career plans, both short- and longterm.

**Methods:**

Purposive sampling of schemes was undertaken to include urban, rural and metropolitan schemes, schemes in areas with and without dental schools and geographic coverage across England and Wales. All VDPs in these schemes were initiated to participate in this qualitative study using focus groups. A topic guide was utilised to standardise data collection. Informants' views were recorded on tape and in field notes. Data were transcribed and analysed using Framework Methodology.

**Results:**

A total of 99 VDPs participated in the 10 focus groups. Their choice of dentistry as a professional career was motivated by multiple categories of influence: 'academic', 'healthcare', 'lifestyle', the influence of 'family', 'friends', 'careers advice' and 'work experience'. Consideration of the features of the 'professional job' appears to have been key to their choice of dentistry and the 'active rejection of medicine' as an alternative career.

Entry into the profession was proving a challenging process for some but not all VDPs. Informants perceived that their vision had been moderated as a result of 'personal student debt', 'national workforce initiatives', 'limitations on clinical practice' and the 'cost of additional training'.

Short term goals focused around 'recovery from the past' and 'preparation for the future'. Longterm goals covered the spectrum of opportunities within dentistry. Factors influencing VDPs longterm career plans fell into six main categories: professional, personal, financial, political, social and cultural.

**Conclusion:**

VDPs chose dentistry because they perceived that it provides a financially lucrative, contained career in healthcare, with professional status, job security and the opportunity to work flexibly. They perceive that their vision is challenged by changes affecting education and the healthcare system. Longterm professional expectations were closely linked with their personal lives and support a vision of a favourable work/life balance.

## Background

### The future workforce

An understanding of the motivation of the healthcare workforce is critical to healthcare systems. In considering the future health workforce, it is recognised that 'health systems which adequately served the limited medical options of the earlier 20^th ^century are unable to cope with modern complexity' [1] and thus will result in major change for the health workforce. The main drivers of workforce change have been categorised as 'technical and scientific developments', 'demography and disease', 'political drivers' and 'wider social and economic change'. These are issues for the healthcare workforce worldwide, albeit that in high- and low income countries, the specifics will differ.

The challenges facing dental professionals have been outlined in detail by Gallagher et al., [2] and involve the above drivers for change. [3] In essence they relate to changing patterns of oral health, [4,5] an ageing population, [6] increased service uptake, [4] science and technological developments relating to dental care, [7] changes in the sex, ethnicity and skill-mix of the dental workforce, [8-12] and major changes in the funding and organisation of dental care systems; [13-17] in a post-modern environment. [18] Since the inception of the National Health Service [NHS], the majority of dentistry in England and Wales, has traditionally been provided within a national system of state funded healthcare, albeit that it has increasingly involved patient charges being levied for most adults. [19] This system has entered a period of fundamental change with the shift to local commissioning of state funded dental care in England and Wales from April 2006. [14-17] At the time of this study, dentistry was on the cusp of this radical policy change. This change has devolved funding to local commissioning organisations which enter into contracts with dental practitioners. These changes will influence professional practice, with implications for workforce flexibility and mobility in both the NHS and private sectors.

### Motivation and the dental workforce

Research over time, and across countries into the motivation of individuals who choose dentistry as a professional career, demonstrates a wide range of motivational factors. [2,20-31] Most of this research has been questionnaire based and has used different instruments; hence the findings are not directly comparable. Whilst there is general agreement on the range of motivating factors, it appears that the dominant motivational factors may vary over time and between countries. Furthermore, from a sociological perspective, there is a growing recognition that the emerging workforce has very different expectations to those of earlier generations, with the greatest influence being generational or 'age-related'. Evidence from the US indicates that these expectations have implications for health profession and the performance of health systems [32-37].

### Motivation of professional groups

Larsson, [38] endorsed by Macdonald [39] has suggested that all professional groups are embarked on a dynamic professional project. Larson, [38] developed a theoretical framework to describe the dynamic nature of professional groups and their need to keep negotiating their professional status in order to continue to hold their elite positions. She defined the overall objectives of the 'professional project' to achieve 'status in the social and economic order' and 'monopoly in the market' for services based on their 'expertise'. Her work [38] was refined by Macdonald. [39] During professional degree studies it is well recognised that students receive both formal and informal training [40]. The latter is an important part of professional socialisation [41,42].

### Professional careers

The literature on professional careers highlights the changing context of careers [43], the need to consider career pathways and career anchors, 'the self image which an individual develops around his or her career which comes to be a guide as well as a constraint on career decisions' [44]. Guest suggests that the concept of the 'psychological contract' is a useful construct for policy and research in a market driven economy where new employment relationships are individualised and inequalities in power exist between individuals and the organisations they work for. [45] The contract is informed by individual's expectations, experience and the organisational climate, policy and practice of the organisation, as well as the possibility of alternatives. If they are met then the consequences may include job satisfaction, motivation organisational commitment; whereas if the psychological contract is not met, the result may be departure from the organisation or workforce [45]. Consideration of these issues is important as retention of the healthcare workforce is recognised as a major challenge problem by planners and policy makers [1,46,47].

### Vocational Dental Practitioners

Between 1995 and 2005 in England and Wales, over 650 dentists enter training annually as vocational dental practitioners, [48] the majority of whom had just graduated from dental school. At regional and local levels their training is under the auspices of postgraduate dental deans/directors and is organised by Regional and (Local) Vocational Training Advisers. There are local training schemes with a day release programme overseen by a Vocational Training Adviser. In 2004/2005, Wales has a total of six Vocational Training Schemes. England has just under 50 schemes, the majority of which start in August/September of each year.

Vocational Dental Practitioners (VDPs) in England and Wales are emerging into a profession and healthcare system undergoing the greatest change since the inception of the National Health Service. To retain a motivated workforce, system reform must be informed by the professional expectations of these new entrants to the profession.

Graduates work as salaried assistants in approved practices with General Dental Practitioners who have been appointed as trainers for a period of one year. During the three academic terms, the VDPs attend an educational day release programme designed to help them understand both the clinical and the administrative aspects of NHS dentistry.

The aim of vocational training is to produce a dentist capable of undertaking independent dental practice. Dentists need to complete Vocational Training before they can practice as a principal in General Dental Services, within the National Health Service. This process is changing with the proposals for foundation training being developed by the Postgraduate Dental Deans [49].

Prior to 2004, the Faculty of General Dental Practice (UK) collaborated with Vocational Training Advisers and VDPs and ran annual VDP national research projects and/or audits, such as those reported by Burke et al., [50] and Palmer and Batchelor. [51] The concept of participation in a national research project was therefore well established within dental vocational training in England and Wales.

The study reported in this paper is part of a wider programme of study to examine student and recent graduates vision of their future professional careers and the key issues they perceive will influence their contribution to dentistry in general and NHS dentistry in particular.

The objectives of this part of the study were to investigate why VDPs' in England and Wales perceive they chose dentistry as a professional career; how they perceive their vision has changed and to explore the implications for their short- and longterm professional expectations. Their views on healthcare delivery systems across the NHS and private sectors, also investigated in the study, will be reported separately.

## Method

### Study design

This study was developed by King's College London Dental Institute (KCLDI) and the Faculty of General Dental Practice (UK) (FGDP) as the VDP project for 2004/05, providing the opportunity for VDPs to reflect on their choice of dentistry both in focus groups and a subsequent questionnaire audit. Ethics committee approval was sought and obtained from King's College London Research Ethics Committee (No. *03/04-111*). Purposive sampling was undertaken to ensure that the groups were representative of the range of VDP training schemes across England and Wales and to achieve a minimum of 10% sample of VDPs to participate in focus groups. Schemes across urban, rural and metropolitan areas, with and without dental schools, whilst also providing geographic representation were selected. VDP Tutors were contacted with details of the study, with the request that their group should be invited to participate during their day release schemes. VDPs were provided with an information sheet relating to the study. Participation by members of the group was voluntary. Informed written consent was obtained prior to commencement of the focus group for those choosing to participate.

### Data collection

A topic guide was utilised to standardise data collection across the groups. Interviews were conducted by three individuals [JG, KE and WC] with a dental public health and behavioural science background. Only the interviewer and VDPs were present during each focus group (interview). At all 10 focus groups the interviewer explored all items on the topic guide. Data were audio-taped and transcribed in preparation for analysis. The study was conducted between September 2004 and April 2005.

### Data analysis

Analysis was undertaken using Framework, a two staged "matrix based method for ordering and synthesising (qualitative) data". [52] It was conducted in Excel building concepts within an analytical framework and using the concepts to develop theory. [53] The process began with familiarisation of the researchers with the raw data in transcription to develop an index or conceptual framework of themes and sub-themes. This index was refined throughout the analysis process as more themes emerge and systematically applied to all of the raw data which was 'indexed' or given a code. This permitted the raw data to be sorted by theme whilst maintaining transparent links with original data. Once the data were sorted they were summarised to reduce the volume, keeping key language, expressions and phrases, and then 'charted' or placed into the relevant part of the framework grid. Next steps involved further abstraction to define the elements and dimensions to a higher level, refine categories and finally classify the data. Finally, explanatory accounts were identified which involved detecting patterns, developing explanations and seeking applications to wider theory. Throughout the analysis the findings were related back to the original transcripts to ensure that the emerging findings accurately reflect the context and views expressed. The data were analysed by two researchers and areas of difference were discussed to achieve agreement.

## Results

### Response

A total of 99 VDPs participated in the 10 focus groups convened across England and Wales during 2004/05. One group had to be replaced because its members were already taking part in workforce related research during this period. In total the participating VDPs represented over one in seven of all dentists in vocational training and achieved geographical coverage across England and Wales across urban and rural, metropolitan areas with and without dental schools. Students represented the range of dental schools in England & Wales, and beyond.

### Motivation

**Figure 1:**
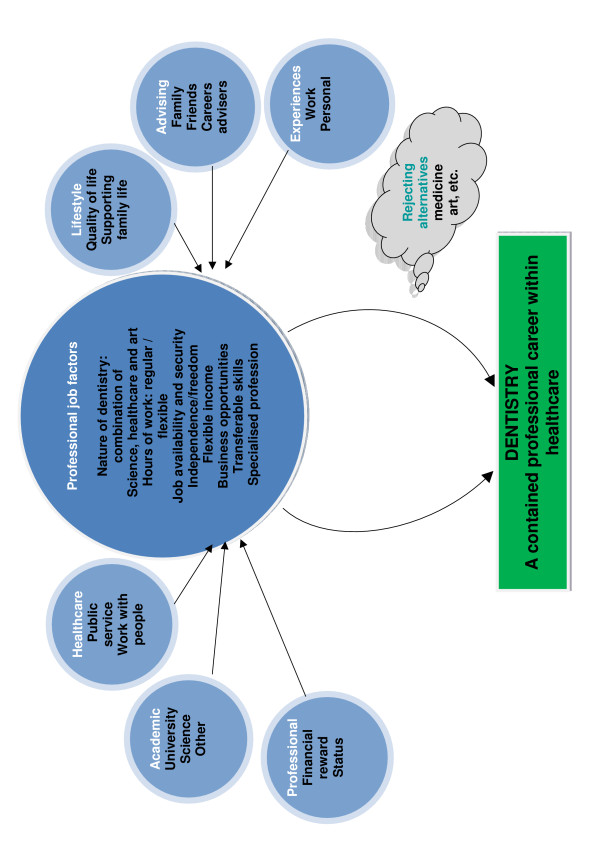
Influences on Vocational Dental Practitioners' motivation for choice of dentistry.

Participants choice of dentistry as a professional career was motivated by multiple categories of influence: 'professional' 'academic'; 'healthcare'; 'lifestyle'; the influence of 'advisors' including family, friends, careers advisers; and 'work experience' as presented in Figure 1. Consideration of the 'features of the professional job' and their influence on 'quality of life' appears to have been central to VDPs choice of dentistry. Features included the nature of dentistry combining science, healthcare and art; the hours of work which can be both regular and flexible; the perception of job availability and security; independence/freedom; and earning a sizeable and flexible income. Dentistry was considered a 'specialised profession' in itself because one could practice without additional qualifications, whilst providing a range of career and business opportunities. It also involved transferable skills. There was a strong sense that the motivating factors had been sifted against the various dimensions of the professional job and found to be acceptable, with many reporting the 'active rejection of medicine' as an alternative career. These issues are reflected in the following quotations:

"(Dentistry provides) flexibility, job security, one of the few professions where you're almost guaranteed a job from university with a healthy salary which is pretty stable throughout your life it you want it to be." Cl68

"It offers a good lifestyle. If you've got [an] interest in science... it's still the medical profession and ... and it's good for women I thought anyway..." K4

### Moving from vision to reality

Moving from 'vision' to 'reality' through their educational experiences and subsequent working practice was proving a challenge for the majority of respondents. Informants perceived that their vision had been moderated as a result of 'personal student debt'; 'national policy initiatives' relating to dental workforce and NHS dentistry. This included the recognition that there were 'limitations on clinical practice', in relation to the location of training and nature of care within the NHS. VDPs were concerned about the challenge of providing preventive care and the 'potential for boredom'. Finally, some VDP**s **raised concerns about the 'cost of additional training', in terms of time, commitment and money and the transferability of their qualification to other international settings. These views are illustrated in the quotations below.

I thought... a professional job where I knew...I'd get paid well. I could afford things that I wanted ... clear the student debt and stuff and ...I never [realised there was a] treadmill system: GREAT! J430

You're really limited in what you can do with patient costs. At university you didn't have any real concerns about that, so that changed a lot of things. J58

... I think that's an issue that they're (government) bringing so many dentists from other countries and we didn't realise when we qualified we'd have to be competing with them for the same jobs. J96

I think ...it's just the way that everything's changing and I'm really sceptical about how it's going to work... B145

I don't know if you'd encourage your kids to go into dentistry, would you? J348

In marked contrast, a few students had discovered that the reality of professional working life was a pleasant surprise preferring the 'real world' to dental school, enjoying the sense of being part of a professional group and being able to practice much more quickly.

I was pretty much forced into doing dentistry, I hated it and I just didn't wanna do it, and I was like failing a year, and all this stuff, now I really really, really love it ... A39

In dental school it's a lot red tape. A filling takes a couple of hours, you know, to do. You don't realise how simple it is until you get out ... then you can start to enjoy it. A44

### Short term goals

Short-terms goals focused around 'recovery from student life' and 'preparation for the future'. Recovery included 'gaining income' to pay-off student debts, 'becoming independent' and 'surviving political changes'. This involved getting a job after completion of vocational training and having a break from the commitments involved with their five or more years of training. Despite their earnings during their VT year, many felt that they were yet to reap the benefits of the time and effort they have put into dental training and wanted to work full time in doing so. Financial reward for their hard work was important and needed to be commensurate with similarly qualified peers. For some dentists, the reality of their financial situation meant that they were re-evaluating professional goals and relinquishing their interest in specialising.

...got quite a lot of debt. Now it's my priority to sort that out. J88

...we've been studying for 5 years...and all our friends have finished their normal degrees and [have] been working for a few years already. They're already on good salaries so... to ... study for another 5 years is ... (too much) (general support from peers) E74

Many of the VDPs were felt that their experiences in the short term would help them in preparation for the future by identifying areas of interest, clarifying their career goals and the types of setting they wanted to work in over the longer term. Many further influences were the same as the longterm issues outlined below...particularly relating to their desired lifestyle.

... I'm not quite sure at the moment... I need to get more experience... I think I'll need about two three years to find my feet and see what I want to do then. D49

Practically, ensuring proximity to family and social networks was considered important, as well as the opportunity to pursue interests outside of dentistry. Meeting family expectations was particularly important for Asian dentists. Cultural expectations differed between males and females, with some females feeling the pressure to live at home until they are married, and some of the males reporting the expectations to provide wider family support.

I just think as an Asian girl... I've been away for some time at Uni... [but] when you've got your job...your parents do have...an expectation that you...live at home until you get married... E314 Female

It makes a difference if you're a boy or a girl as well. It makes it difficult because I mean if you've been at Uni for five years and you're a boy, your family would expect you to now start earning and contributing to the family – family unit becomes stronger... E321 Male

### Long term goals

**Figure 2:**
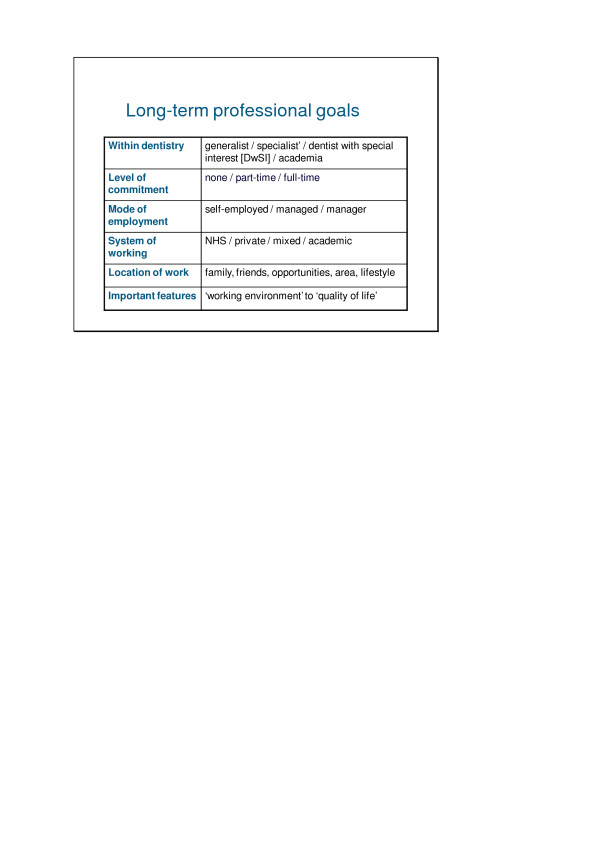
Long-term professional goals: spectrum of issues.

Longterm professional goals covered the scope of the profession as outlined in Figure 2. They included: exploring career opportunities from 'generalist' to 'specialist'; location of work; modes of employment; systems of working; levels of commitment; working environment and quality of life. The geographical location was likely to be determined by a wide range of factors, the most important of which appeared to be family, career opportunities, interests outside of work, the type of area and lifestyle goals.

### Influencing long term goals

**Figure 3:**
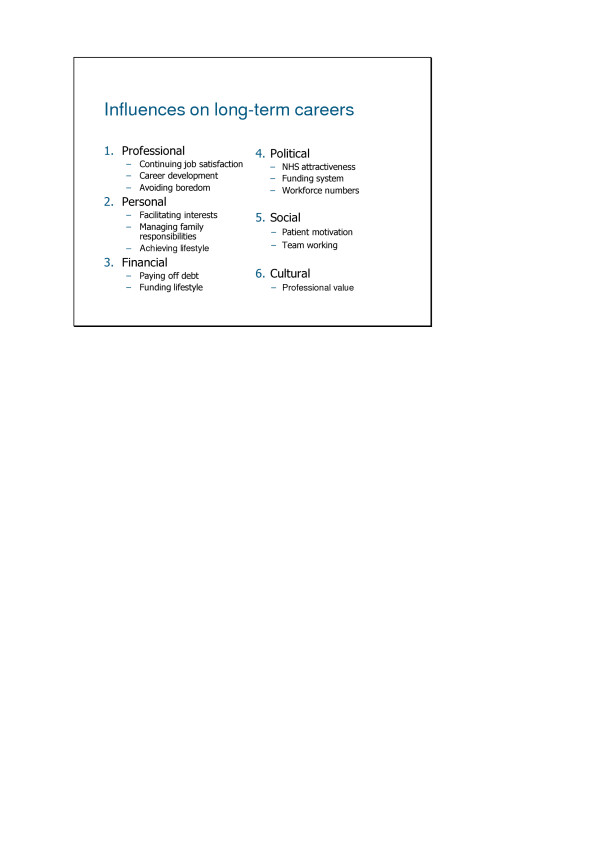
Influences on long-term careers reported by Vocational Dental Practitioners in England and Wales.

Factors influencing VDPs longterm career plans fell into six main categories: 'professional', 'personal', 'financial', 'political', 'social' and 'cultural', (Figure 3) each of which will be explored in detail, with supporting quotations.

#### Professional

Professional factors included achieving 'continuing job satisfaction' and 'career development', thus 'avoiding boredom'.

Continuing job satisfaction throughout their career was an important factor for many VDPs and would determine their longer term contribution to dentistry. This was associated with the extent of their workload, in terms of patient numbers, dental materials and time.

"Job satisfaction – having the time to do the work you were trained to do. I get frustrated when I can't do something but you know you can do something but because your limited by time and materials as well." K106

The ability to expand and develop in their careers in the long term, giving them goals to work towards was also considered an important. This included developing the business side of dentistry, with or without career development options. Many of the VDPs did not want to return to work in a hospital setting to be able to specialise, because of the financial implications of doing so and the time commitments involved. They considered becoming a 'dentist with a special interest' as attractive, providing the best of both the generalist and specialist world. This involved developing skills beyond that of a generalist, at a later stage in their professional career when they had achieved some financial stability, thus enabling them to avoid possible boredom of repetitive work.

"I think it (becoming a dentist with a special interest) will be very attractive to dentists in general because...it's a long route to go ... to be on the specialist register...and [if/when]...you decide you like something, you might feel that it's already too late to go back to the hospital and do it... your options in that case are directed to 'special interest'..."E103

"Yes exactly, and that [DwSI] stimulates interest as well ...stops it becoming boring..." J501

#### Personal

The key dimensions of the personal category are 'managing family responsibilities'

and 'achieving lifestyle'. Family responsibilities related to having children, with both males and females expressing a desire to fulfil family responsibilities.

"I'd like to work four days a week, then I'd like to have children, and possibly work three days a week from quite an early stage. I don't ever want to be full time. B79 Female

...mainly family [will influence me] to be honest so I'll probably... work to comfortable level. I'd rather have that little bit less money in the bank but be able to have time to spend... [with family] C270 Male

The ability to achieve and maintain their desired lifestyle, while working the hours of choice, and developing a career in dentistry had been major factors in their choice of career and were perceived as central to their longterm career choices. There was a strong view that their career was not the principal focus of life and the desire to minimise stress and achieve a balance between work and life was important.

"Life's too short" [to work all the time] J260

"...you don't want to become a boring person; you want to have balance and...a good lifestyle, as well as having a work commitment as well." J261

We all want a good quality of life. C340

Flexibility to take time out if you want to... K142

#### Financial

Financial considerations were strongly related to 'professional' and lifestyle factors. The VDPs were keen to earn an income sufficient to support a good lifestyle, allowing them to work less than full time hours, support their family, and perhaps retire early. Ensuring that they earned a good income commensurate with other similar professionals that repaid their commitment to their training, student debt and support from family was an important factor that would influence the VDPs across their careers. Changes to the system of remuneration and support to pursue further training were likely to impact on this. However, not all VDPs were concerned about earning more than was required to be 'comfortable', being willing to trade money for time as part of their desired lifestyle.

"Basically, the cost of my lifestyle is going to determine where I work so ... if I can work on the NHS for my lifestyle than that's fine but if I can't, I won't." D139

...it's a job and I'll do it as a job. It's not the main focus of my life and it's something I'll do around having kids and being married and I'll do it until I'm rich enough to retire and travel the world. B187

#### Political

Concerns were raised about the impact of policy changes, both in terms of the impending new dental contract, system of remuneration and international recruitment. [54] A few of the VDPs raised concerns about the impact of overseas recruitment of dentists on the business of dentistry including wages, work conditions and job availability in the long-term.

"I think that's an issue that they're bringing so many dentists from other countries and we didn't realise when we qualified we'd have to be competing with them for the same jobs." J96

#### Social

Many of the VDPs emphasised the importance of social factors on day to day working, stating a preference for larger teams or practices. Larger team practices were associated with a more social environment, greater levels of support and easy access to advice, as well as being likely to have better resources and opportunities. The skill mix offered by larger practices was also considered valuable. The VDPs were generally positive about the inclusion of Dental Care Professionals and their future practice.

"I think a multidisciplinary practice....dentists with special interest so you deal with a problem you can always ask an associate, they're always close by for consultation, so you can ...talk about it over lunch and discuss any issues." E132

"It's good being part of a skill mix team...it's very good, 'cause not only... can ideas flow between the team, but also it makes you...feel part of something, when you're part of a bigger system." J413

#### Culture/Professional value

Professional standing and respect for dental professionals from peers, government and the general public was particularly important to VDPs. They wanted some acknowledgement that they had worked hard to get to their position. The VDPs felt that many patients only visited the dentist when they had a problem or in an emergency, were not interested in prevention and placed greater priority on the rest of their body rather than on their oral health. They also felt that in general, dentistry and dentists are undervalued and treated accordingly. One particular concern raised by Asian students was the issue of giving advice to their elders, which could be a constraint to professional practice when newly qualified.

"...You can really get frustrated with patients who just don't listen and don't, just aren't dentally motivated." K189

The media have a role to play in it. You know they highlight all the negative things...they don't come into the practice and see what W, Y and Z does every day of the week, 52 weeks of the year or 40 hours. It's always the negatives you hear, you never hear about the positives. K167

...apart from everything else, if I ever felt like what I was doing wasn't being appreciated then I'd pack it [dentistry] in definitely. B 204

### Finally..

The major reasons for considering leaving dentistry in the future appeared to include demotivation due to stress, threat of litigation, high workloads, poor pay and boredom from repetitive, unrewarding and routine work, and feeling undervalued. Whereas professional stimulation with interesting and varied work, good financial rewards and have the type of lifestyle they want were central to a positive vision of their future working lives.

## Discussion

### Benefits and limitations of the study

Although there have previously been a series of national VDP projects in England and Wales, [50,51] they have involved VDPs as researchers. The study reported in this paper is the first national qualitative study of VDPs to explore their professional motivation and the issues which new entrants to primary dental care perceive will influence their future careers. Past studies have examined VDPs preparedness for entry into training, [55] and their training experiences. [56] The use of a qualitative approach provided the opportunity to explore the range of issues pertinent to this cohort of dentists and gain insight into perceptions and beliefs [57-59]. Using focus groups where the participants who are known to each other, as with these VDP tutor groups is recognised to have added benefits. [57] These include the ability to share and discuss ideas, attitudes and common experiences, allowing exploration of shared meanings and contexts. However, the disadvantage of using groups where the participants are known to one another is that shared assumptions are made and therefore some issues may not be explored fully. Furthermore, non- or limited participation may mean that more extreme views are not shared. The method generally worked well with VDPs who quickly entered into dialogue and proved ready to share their views including diverse perspectives. The data were becoming saturated from the midpoint of the study onwards; however, focus groups were continued in line with purposive sampling to achieve geographic coverage.

The timing of the study was such that the detailed arrangements for the new dental contract in England and Wales were under discussion. The views expressed here therefore relate to a context of professional uncertainty in the midst of policy change. These included a period of international recruitment, and high demand for the Overseas Registration Examination (formerly the International Qualifying Exam). Whilst the policy climate may have created uncertainty about the future, it may have helped to focus the minds of these dentists on what they wanted from their professional career. However, at time of writing there is continuing uncertainty and possibly looming unemployment. Expanded undergraduate numbers are putting pressure on the number of VT places and will increase competition for jobs, unless there is a significant increase in NHS and/or private funding. Consideration will be given to repeating this study when new arrangements become established.

### Short term professional careers

By the time VDPs participated in the present study, they had been working in the real world (i.e. away from their dental schools) for several months or more, albeit in a semi-protected environment. For some this was proving a pleasant surprise, whilst for others the challenges of professional life were just becoming apparent. This is a seminal point in a professional career as young dentists begin 'recovery from student life' and embark on 'preparation for the future'. Clearly the long undergraduate programme has taken its toll financially and this influences career decisions. This has been recognised for some time, [2,60] but never addressed constructively in health or academic policy across England and Wales.

A prolonged 'recovery from student life' possibly out of financial necessity appeared to influence attitudes to further training and, in turn, limit options in respect of 'preparation for the future'. This has implications for the level of specialist expertise within the profession, many of whom are close to retirement. [61]

Proposals in respect of UK Dental Foundation Training will be very important [49] and need to ensure that they are relevant to prepare young dentists for future professional life. A structured two year programme will facilitate broad professional development, provided it takes account of the needs of new graduates. One example arising from this study relates to location. As the intake of dental students currently involves a high proportion of Asians, [11] and females, [12] it will be important to consider the location of general professional training so that they can fulfil the cultural family responsibilities as highlighted in this study. Such considerations need to be balanced with geographic variations in dental workforce needs.

A further development may provide additional career flexibility. It is the creation of the Membership of the Joint Dental Faculties (MJDF) by the Faculties of Dental Surgery and General Dental Practice of the Royal College of Surgeons of England. [62] This replaces two different Memberships, one leading to specialist training which necessitated a period in a recognised hospital post and the other which formed part of professional development for primary dental care practitioners. This single qualification, which can become a desirable requirement for higher specialist training, will give far greater flexibility to young dental graduates in their career planning. Together with the recent proposals from the General Dental Council regarding specialist training, [63] this should facilitate a no less demanding but more flexible entry process for those who wish to undertake specialist training, perhaps at a later stage in their professional career.

### Motivation for choice of dentistry and long term careers

The results of this study support the findings of Gallagher et al. amongst final year dental students at KCLDI, [2] confirming the wide range of influences on this generational cohort's choice of career, lending support to the range of influences raised in similar studies of applicants to or students of dentistry. [24,26-28,30,64,65] Qualitative research supported the view that features of the professional job were important concluding that the underlying motivation for dentistry was the desire for a 'professionally contained career' in healthcare, attracted by aspects of the job which they perceive offer security, flexibility and quality of life [66]. Triangulation of these findings with a subsequent quantitative study of final year students lent further support to the range of motivational factors in the choice of dentistry and the dominance of 'professional job' factors. [67] This is clearly demonstrated in the 'active rejection' of medicine amongst VDPs in this study and undergraduates at KCLDI, [2] as a profession of choice due to certain aspects of the professional job. The dangers of a workforce being attracted by 'features of the professional job' which are all liable to change during their working lives' if not by the time they leave college, have already been discussed by Gallagher et al. [2]

Students were quick to highlight that their vision of professional working lives had changed, and now included a range of constraining influences; these related to national policy initiatives such as international recruitment, [54,68-70] which may threaten job security, and student debts which pose a challenge to financial stability [60,71].

Motivation for the VDPs choice of a dental career was strongly related to the issues which they considered would influence their longer term careers. Professional, personal and financial factors were interlinked and considered particularly important. As with their choice of career, there was a sense of filtering professional opportunities against their personal and financial goals. The emphasis on 'quality of life' and achieving a favourable 'work/life' balance as motivating factors for choosing dentistry, was marked. Similar findings are evident from a recent survey of US dental students across schools, [26] and King's College London Dental Institute. [66] This is possibly evidence of the generational effect whereby this age cohort of graduates hold specific priorities [32-36].

### Implications for professional groups and policy makers

These findings provide support to sociological analyses of the generational effect in the workplace whereby different age cohorts have different workplace and lifestyle characteristics, social values and motivation. They support the view that young people emerging into the workforce in general 'netsters' place much greater emphasis on 'lifestyle' when compared with senior colleagues 'baby boomers', in the workplace, [32-36] including healthcare. [37] However, financial motivation amongst dental 'netsters' would appeared to receive greater prominence than sociologists would suggest for this generation in the workplace [34-36].

Moats Kennedy's theory that 'lifestyle' is of prime importance and that time off may be more important than money, with mentoring, meeting own goals and training considered important rewards. [34-36] If their goal is a 'professionally contained career in healthcare', with the additional trappings of money, job security and flexibility and the desire to 'do well, before they do good', this needs to be recognised. [34-36] Altogether, the findings of this qualitative study appear to complement quantitative research in this field and identify the underlying issues influencing current graduates' choice of professional career and long term expectations. The findings would suggest that there is a series of future implications which need to be thought through by policy makers and professional leadership as they relate to workforce motivation, workforce capacity, health and education policy and continuing professional development. To retain a motivated dental workforce, future system reform must be informed by an understanding of the current generational cohort and arrangements to meet, and thus retain, the professional expectations of new entrants to the profession.

Of course, we cannot test the validity of response and it will be important to monitor the activity of this cohort over time.

## Conclusion

VDPs chose dentistry because they perceived that it provides a financially lucrative, contained career in healthcare, with professional status, job security and the opportunity to work flexibly. They perceive that their vision is challenged by changes affecting education and healthcare, with implications for their future motivation. The longterm professional expectations of VDPs are closely linked with a vision of a favourable work/life balance.

## Competing interests

Two of the authors (JEG and NHFW) are academic staff at King's College Dental Institute, one of whom (NHFW) is Dean and Head of School. KAE works for the Faculty of General Dental Practice (UK) as Editor of the journal Primary Dental Care and a National Research Facilitator. KAE and NHFW are members of the Faculty's Research Committee.

## Authors' contributions

JEG, KAE and NHFW conceived and designed the overall research programme. JEG led the development of the protocol, gained ethics committee approval and led on writing of the paper. JEG, WC and KAE conducted the fieldwork. JEG and WC undertook framework analysis. JEG, KAE and NHFW contributed to the paper. All authors reviewed the final manuscript.

## Pre-publication history

The pre-publication history for this paper can be accessed here:


